# Challenges and standardization of microRNA profiling in serum and cerebrospinal fluid in dogs suffering from non-infectious inflammatory CNS disease

**DOI:** 10.1186/s13028-019-0492-y

**Published:** 2019-12-03

**Authors:** Susanna Cirera, Emilie Ulrikka Andersen-Ranberg, Sille Langkilde, Maria Aaquist, Hanne Gredal

**Affiliations:** 1 Department of Veterinary and Animal Sciences, Faculty of Health and Medical Sciences, University of Copenhagen, Grønnegårdsvej 3, 2nd Floor, 1870 Frederiksberg C, Denmark; 2 Department of Veterinary Clinical Sciences, Faculty of Health and Medical Sciences, University of Copenhagen, Dyrlægevej 16, 1870 Frederiksberg C, Denmark

**Keywords:** Biomarker, Cerebrospinal fluid, Circulating, CNS, MicroRNA, MUO, qPCR, Serum, SRMA

## Abstract

Non-infectious inflammatory (NII) central nervous system (CNS) conditions are primarily diagnosed by the demonstration of inflammatory changes in the cerebrospinal fluid (CSF). However, less-invasive methods and peripheral biomarkers are desired. Changes in circulating microRNA (miRNA), which are short non-coding regulatory RNAs, may serve as biomarkers of disease. The aim of this pilot study was to investigate selected miRNAs in serum and CSF, hypothesizing that the levels of specific miRNAs in serum correlate with their presence in CSF, and that changes in serum miRNAs levels may reflect CNS disease. We profiled serum and CSF samples using quantitative real-time PCR (qPCR) searching for selected and previously profiled miRNAs in serum (let-7a, let-7c, miR-15b, miR-16, miR-21, miR-23a, miR-24, miR-26a, miR-146a, miR-155, miR-181c and miR-221-3p) and in CSF (let-7c, miR-16, miR-21, miR-24, miR-146a, miR-155, miR-181c and miR-221-3p) from 13 dogs with NII CNS disease and six control dogs. We demonstrated the presence of several miRNAs in CSF (let-7c and miR-21 dominating) and serum (miR-23a and miR-21 dominating). However, we generally failed to reproduce consistent results in CSF samples due to several reasons: unacceptable PCR efficiency, a wide variation between cDNA replicates and/or no-amplification in qPCR suggesting very low levels of the investigated miRNAs in canine CSF. Serum samples performed better, and 10 miRNAs qPCR assays were qualified for analysis. We were nevertheless unable to detect a difference in the expression of miRNA levels between cases and controls. Moreover, we could not confirm the results of recent miRNA investigations of canine CNS diseases. We believe that these disagreements highlight the significant effect of methodological/analytical variation, rather than the incapacity of circulating miRNAs as biomarkers of CNS disease. A secondary aim was therefore to communicate methodological challenges in our study and to suggest recommendations for circulating miRNA profiling, including pre-, post- and analytical methods based on our experience, in order to reach reproducible and comparable results in veterinary miRNA research.

## Findings

Non-infectious inflammatory (NII) central nervous system (CNS) conditions, including steroid responsive meningitis-arteritis (SRMA) and meningoencephalitis of unknown origin (MUO), are common causes of severe neurological disease in dogs. A diagnosis is primarily based on cerebrospinal fluid (CSF) analysis. However, as CSF sampling is associated with some risk and requires general anesthesia, less-invasive methods and biomarkers are desired. MicroRNAs (miRNAs) are short non-coding RNAs, which play an important role in gene regulation of many physiological and pathological processes [1]. Changes in circulating miRNA may therefore potentially serve as biomarkers of disease offering an opportunity to study disease processes by minimally invasive methods.

In veterinary medicine, miRNA profiling is still in its infancy, and most studies concerning miRNAs involve tissue or serum samples. While miRNA profiling in CSF has been successfully conducted in humans [2–5], to our knowledge only three studies in dogs are available [6–8].

The overall aim of this pilot study was to investigate the relative expression of selected miRNAs in serum and CSF from dogs with NII CNS disease in comparison with non-affected dogs in order to evaluate the potential of miRNAs in serum as less-invasive biomarkers than existing diagnostic methods. Our hypothesis was that the levels of specific miRNAs in serum correlate with their levels in CSF and thus can reflect CNS inflammation. However, while conducting this work, we faced several methodological challenges. A secondary aim was therefore, based on our experience, to suggest best-practice methods for future studies of miRNA profiling in dogs.

We conducted a prospective study of dogs with NII CNS diseases. Thirteen dogs (eight females and five males) with NII CNS disease (seven with MUO and six with SRMA) were included in the study (exclusion and inclusion criteria are detailed in Additional file 1). In addition, six control dogs with no signs of neurological or systemic inflammatory disease were included (two females and four males) amongst dogs presented for euthanasia at the University Hospital for Companion Animals, University of Copenhagen (Additional file 1, Table 1).

**Table 1:** Dogs included in the analysis, MUO (n = 7), (SRMA) (n = 6), and controls with no signs of systemic or neurological disease (n = 6)

Dog id	Diagnosis	Group	Breed	Age	Sex
1	MUO	Affected	Boxer	5 years	F
2	MUO	Affected	Chihuahua (shorthaired)	2 years 10 months	F
3	MUO	Affected	Weimaraner	8 years 3 months	M
4	MUO	Affected	Chihuahua mix	7 years 9 months	M
5	MUO	Affected	Chihuahua (shorthaired)	3 years 5 months	F
6	MUO	Affected	Border collie	7 years 5 months	F
7	MUO	Affected	Cairn terrier	7 years 1 months	F
8	SRMA	Affected	Shih szu	1 years	F
9	SRMA	Affected	Chesapeake bay retriever	7 months	F
10	SRMA	Affected	Stabyhoun	1 years 11 months	M
11	SRMA	Affected	Boxer	1 years 9 months	M
12	SRMA	Affected	Flat coated retriever	1 years 5 months	M
13	SRMA	Affected	Japanese akita	1 years 2 months	F
14	Chronic osteo-arthritis, blindness	Control	Welsh corgi cardigan	12 years 4 months	Mn
15	Behavioral	Control	Bull terrier	3 years 8 months	Mn
16	Non spinal back pain	Control	Rottweiler	7 years	M
17	Perianal tumor	Control	Miniature pinscher	13 years 4 months	Fn
18	Behavioural	Control	Mixed medium breed	1 years 2 months	F
19	Chronic osteo-arthritis	Control	Bull terrier	7 years 3 months	M

*MUO* meningoencephalitis of unknown origin, *SRMA* steroid-responsive meningitis arteritis, *F* female, *Fn* female neutered, *M* male, *Mn* male neutered

Blood and CSF were collected from each animal (Additional file 1) and centrifuged at 2000*g*, 4 °C, for 15 min to eliminate cellular debris. Serum samples were allowed to clot for 15 min prior to centrifugation. Samples were visually inspected for presence of hemolysis. Supernatants were aliquoted (200 μL) into 1.5 mL RNase-free cryo-tubes and frozen at − 80 °C within 2 h of collection.

Total cell-free RNA, including miRNAs, was purified from serum and CSF samples using the “miRNeasy serum/plasma Kit” (Qiagen, Hilden, Germany) following the manufacturer’s protocol with the single modification of adding MS2 phage RNA carrier (Roche Diagnostics, Hvidovre, Denmark) to the QIAzol lysis reagent (Qiagen, Hilden, Germany) at a concentration of 1.2 μg/1 mL, as recommended by Enelund et al. [9] and Andreasen et al. [10] to improve the amount of RNA recovered. Nanodrop spectrophotometer (ThermoScientific, Hvidovre, Denmark) was used to evaluate RNA quantity and quality.

Two and three individual cDNA syntheses were made from each RNA stock of CSF and serum samples, respectively. 1 μL of each RNA stock was used for cDNA synthesis according to Balcells et al. [11]. A synthetic miRNA from *Caenorhabditis elegans* (Cel-miR-39a) was added as a spike-into each sample for cDNA synthesis. Samples were diluted eight-fold prior to qPCR processing. Profiled miRNAs were selected based on relevant published studies (Table 2, Additional file 1). Primers (Table 2) were designed using the software “miR-primer” [12]. QPCR was performed on a MX3005P system (Agilent Technologies, Glostrup, Denmark) following the protocol used by Enelund et al. [9] (see details in Additional file 1). Raw qPCR data (Additional file 2) were initially curated manually according to guidelines detailed in Additional file 1. Subsequently, data were processed using GenEx Pro (Multid Analyses AB, Gothenburg, Sweden) including efficiency correction, normalization, averaging cDNA replicates, fold change calculations between affected and control dogs and log2 transformation. A two-sided non-parametric Mann–Whitney test was used for statistical comparison. P values were corrected for multiple testing.

**Table 2:** Mature sequences and forward and reverse primers for each microRNA tested

Name	Mature sequence	Forward primer	Reverse primer
let-7a	UGAGGUAGUAGGUUGUAUAGUU	GCAGTGAGGTAGTAGGTTGT	GGTCCAGTTTTTTTTTTTTTTTAACTATAC
let-7c	UGAGGUAGUAGGUUGUAUGGUU	GCAGTGAGGTAGTAGGTTGT	GGTCCAGTTTTTTTTTTTTTTTAACCA
miR-15b	UAGCAGCACAUCAUGGUUUACA	GCAGTAGCAGCACATCA	GGTCCAGTTTTTTTTTTTTTTTGTAA
miR-16	UAGCAGCACGUAAAUAUUGGCG	CAGTAGCAGCACGTAAATATTG	CAGTTTTTTTTTTTTTTTCGCCAA
miR-21	UAGCUUAUCAGACUGAUGUUGA	TCAGTAGCTTATCAGACTGATG	CGTCCAGTTTTTTTTTTTTTTTCAAC
miR-23a	AUCACAUUGCCAGGGAUUU	AGATCACATTGCCAGGGA	GGTCCAGTTTTTTTTTTTTTTTAAATCC
miR-24	UGGCUCAGUUCAGCAGGAACAGG	AGTGGCTCAGTTCAGCA	CCAGTTTTTTTTTTTTTTTCCTGTTC
miR-26a	UUCAAGUAAUCCAGGAUAGGCU	GCAGTTCAAGTAATCCAGGATAG	GTCCAGTTTTTTTTTTTTTTTAGCCT
miR-146a	UGAGAACUGAAUUCCAUGGGUU	CAGTGAGAACTGAATTCCATG	GGTCCAGTTTTTTTTTTTTTTTAACC
miR-155	UUAAUGCUAAUCGUGAUAGGGGU	CGCAGTTAATGCTAATCGTGATAG	AGGTCCAGTTTTTTTTTTTTTTTACC
miR-181c	AACAUUCAACCUGUCGGUGAGUU	GAACATTCAACCTGTCGGT	GGTCCAGTTTTTTTTTTTTTTTAACTCA
miR-221-3p	AGCUACAUUGUCUGCUGGGUUU	CAGAGCTACATTGTCTGCTG	TCCAGTTTTTTTTTTTTTTTAAACCCA
Cel-miR-39a	UCACCGGGUGUAAAUCAGCUUG	GTCACCGGGTGTAAATCAG	CCAGTTTTTTTTTTTTTTTCAAGCTG

For the CSF samples, four assays of the total eight tested did not result in PCR efficiency within the acceptable range of 80–110%. Moreover, all miRNA assays showed inconsistency between some of the cDNA replicates and had samples that did not amplify or had values below the limit of detection (LOD) (Cq values > 34 cycles). Therefore, data from CSF samples were excluded from further analysis. On visual assessment let-7c and miR-21 showed the highest expression in CSF.

Regarding serum samples, one assay was discarded (miR-26a) due to lack of specificity in the melting curve and one assay (miR-221) was below LOD. In summary, expression data for 11 (including assay for Cel-miR-39a) of the serum assays qualified for further analysis. MiR-23a and miR-21 were found to be the most expressed miRNAs in serum (all samples with Cq < 30).

In order to identify stable reference miRNAs for normalization, we used GeNorm [13] and NormFinder [14]. These programs rendered different results, i.e. GeNorm identified let-7c and let-7a as feasible normalizers (stable), and NormFinder, miR-15b. We trialed both options but did not find any significant differential expression for any of the assayed miRNAs.

Due to the lack of statistical significance in serum analyses and the levels of miRNAs in CSF being too low for quantification, we were unable to investigate a possible correlation between serum and CSF for the studied miRNAs. Moreover, we were unable to support the findings of similar miRNA studies in dogs, which have found higher levels of miR-21 and miR-181c in CSF of dogs with MUO compared to dogs with other neurological disorders [6]. We believe that difficulties in replicating results between studies in the field of circulating miRNAs, rely significantly on the compelling effect of methodological variation on results, rather than the incapacity of miRNAs as biomarkers of CNS diseases. This emphasizes the need for standardized methods in miRNA profiling, and there are other studies pointing in the same direction [15, 16].

Our methodological recommendations, based on experience from the present and previous studies, are described below and summarized in Table 3.

**Table 3:** A summary of the authors’ recommendations for standardized methods in circulating miRNA profiling

Stage	Method	Recommendation
Pre-analytical	Sampling	Freeze samples at − 80 °C (or at least − 20 °C) as soon as possible within a standardized time range for all samples. We suggest within 1 h
Pre-analytical	Centrifugation	Centrifuge samples to eliminate circulating cells or debris under standardized settings (speed, temperature), using the same centrifuge if possible. We suggest 2000*g*, at 20–25 °C for 15 min
Pre-analytical	Hemolysis detection	Inspect presence of hemolysis by spectrophotometric absorbance at 414 nm, or by monitoring qPCR miR ratio between miR23a and miR 451a
Pre-analytical	Carrier	Use a carrier during RNA extraction, for fluids expected to contain low levels of miRNAs (such CSF, serum, urine). We suggest MS2 phage RNA carrier
Pre-analytical	cDNA synthesis	Perform 2–3 replicates for each RNA stock to detect possible inhibitors carried over from RNA isolation
Pre-analytical	Spike-in	Use an exogenous miRNAs (e.g. Cel-miR-39a) to add prior to RNA isolation or cDNA synthesis to assess technical performance
Pre-analytical	Primers	Re-design primers with PCR efficiencies outside the range of 80–110%
Analytical	Normalization	For normalization, several miRNAs should be tested for stability between controls and disease samples using suitable software algorithms, for example GeNorm and/or NormFinder. We recommend using two or more miRNAs for normalization if possible
Analytical	Statistics	Normal distributed data: use parametric test (e.g. t-test, ANOVA) Data not normal distributed: use non-parametric test (e.g. Mann–Whitney test) Apply more complex model (seek professional statistical assistance) if the model has several confounding variables (gender, age, etc.) Correct P values for multiple testing

We have previously shown that different miRNAs degrade at different pace according to their sequence [9, 17]. Accordingly, it is crucial that the processing of all samples follow a strict protocol to ensure reproducibility. We recommend freezing samples at − 80 °C as soon as possible and within a standardized time frame. In the present study, CSF and serum samples were frozen at − 80 °C within 2 h. However, most of the studies focusing on circulating miRNAs do not report the time from sampling to freezing, which is important in order to evaluate the credibility of the results and to ensure reproducibility.

Centrifugation to eliminate cells and debris should be performed at the exact same speed, temperature and equipment, if possible. We were unable to find a consensus of centrifugation speeds and times in previous literature but decided to follow the centrifugation procedure of Sørensen et al. [3] based on their repeated successful miRNA screening in CSF.

In the present study we assessed hemolysis by visual inspection only. Nevertheless, there are technical methods available, which are more accurate, e.g. spectrophotometric absorbance at 414 nm (absorbance peak of free hemoglobin) or a miR ratio between different affected/unaffected miRNAs (e.g. miR-451a and miR-23a-3p) as proposed by Blondal et al. [18].

Several commercial methods for the isolation of (small) RNAs from body fluids are available. Our methods of choice, based on previous experience [9, 19] was the miRNeasy kit (Qiagen, Hilden, Germany). Moreover, we previously assessed (data not shown) that miRNA recovery improves considerably with the addition of carrier RNA in the lysis buffer, in agreement with other studies [9, 10]. We strongly recommend the use of carrier during RNA extraction, especially from fluids expected to contain low levels of miRNAs such as CSF, serum or urine.

Reverse transcriptase is highly sensitive to contaminants and the cDNA synthesis efficiency varies greatly depending on the purity of the RNA samples. Due to the challenges with normalization, potential problems with the cDNA synthesis must be investigated by performing 2–3 cDNA replicates for each RNA sample, accepting only a standard deviation (SD) between Cq values of the replicates of < 1. In the present study, the SD between replicates of cDNA synthesis from CSF was > 1, and data were therefore excluded from further analysis. Unfortunately, previous studies of miRNA profiling in dogs do not report technical repeats at the levels of cDNA; the reproducibility of these results can therefore not be assessed [6, 7].

Data correction by PCR efficiency is often not reported in miRNA profiling studies. Primers performing sub or supra-optimally (PCR efficiency out of 80–110% range) will thereby likely lead to faulty conclusions and should be re-designed.

A gold standard normalization strategy in profiling circulating miRNAs is still lacking [15]. Many studies use the spike-in miRNAs for normalization [8], although it is known that synthetic RNA spike-ins do not reveal the RNA content and quality of the biological sample and should only be used for the identification of technical outliers (see recommendations at http://www.exiqon.com/ls/Documents/Scientific/PCR-spike-in-manual.pdf). According to MIQE guidelines [20], the most correct way to normalize miRNA qPCR data is using two or more stably expressed endogenous miRNAs. Standard algorithms to check stability of miRNAs using different mathematical approaches are available, however, these often yield different results. The choice of normalizer is therefore a subjective decision, which can influence the results as in the present study.

In conclusion, we were unable to reproduce the results from recent veterinary studies of miRNAs in CNS diseases, and to demonstrate a correlation between the selected miRNA concentrations in serum and CSF of dogs with NII CNS disease. However, we believe in the potential of miRNAs as biomarkers of NII CNS disease, and therefore encourage future research to follow a standardized methodology for both preanalytical and analytical steps. We suggest recommendations for best practice based on our experience to benefit future miRNA profiling in veterinary medicine.

## Supplementary information

**Additional file 1.** Details on patient recruitment, sample collection, profiled miRNAs, qPCR protocol, manual curation of qPCR data, including relevant references.

**Additional file 2.** Raw qPCR data from serum samples 1 and 2 are the two cDNA replicates; NTC, sample without template; No RT, sample done without PAP polymerase. The assay miR-221-3p was out of LOD.

## Data Availability

The datasets used and/or analyzed during the current study are available from the corresponding author on reasonable request.

## Abbreviations

CNS: central nervous system
Cq: quantification cycle
CSF: cerebrospinal fluid
LOD: limit of detection
MIQE: minimum information for publication of quantitative real-time PCR experiments
miRNA: microRNA
MUO: meningoencephalitis of unknown origin
NII: non-infectious inflammatory
qPCR: quantitative real-time PCR
SRMA: steroid responsive meningitis-arteritis
