# Supplementary material for: Challenges and standardization of microRNA profiling in serum and cerebrospinal fluid in dogs suffering from non-infectious inflammatory CNS disease
Source: Acta Vet Scand. 2019 Dec 3;61:57. doi: 10.1186/s13028-019-0492-y (PMC6889416; doi:10.1186/s13028-019-0492-y)
Supplement: Supplementary file 1 — Additional file 1. Details on patient recruitment, sample collection, profiled miRNAs, qPCR protocol, manual curation of qPCR data, including relevant references. [file 13028_2019_492_MOESM1_ESM.docx]

**Additional file 1** Details on patient recruitment, sample collection, profiled miRNAs, qPCR protocol, manual curation of qPCR data, including relevant references

***Patient recruitment and screening***

All dogs were recruited from the University Hospital for Companion Animals, University of Copenhagen. The study was approved by The Local Administrative and Ethics Committee. Written consent was obtained from all owners.

Diseased dogs: A total of 13 dogs (eight females and five males) with non-infectious inflammatory central nervous system (CNS) disease, seven with meningoencephalitides of unknown origin (MUO) and six with steroid responsive meningitis arteritis (SRMA) were included in the study. Inclusion criteria for dogs with non-infectious inflammatory CNS disease (SRMA or MUO) were: 1) clinical and neurological signs suggestive of inflammatory CNS disease confirmed by a total nucleated cell count (TNCC) >5 cells/μL in cerebrospinal fluid (CSF) including characteristic cytological findings, and 2) support of the clinical suspicion by magnetic resonance imaging (MRI) when available. Dogs under immuno-suppressive treatment or with other concurrent CNS disease were excluded. Age ranged from 1 – 7 years.

Control dogs: A control population (n=6) was included amongst dogs presenting at the University Hospital for euthanasia. Inclusion criteria were; healthy dogs or dogs diagnosed with a well-characterized non-neurological, non-systemic disease. Exclusion criteria were; signs of systemic inflammation (based on serum c-reactive protein (CRP) > 25 mg/L), or signs of CNS disease based on the dog’s medical history, clinical examination and TNCC >5 cell/µL in CSF analysis. The group of control dogs ultimately included two healthy dogs (euthanized due to behavioural problems), two dogs with chronic osteoarthritis, one with backpain and one with a perianal tumor. Age ranged from 1 – 13 years.

***Sample collection***

Blood was collected by venipuncture from either jugular-, cephalic- or saphenous vein into serum tubes using BD vacutainer system (Greiner Bio-One, Denmark). Cerebrospinal fluid was collected from the cerebello-medullary cistern with the dog placed in lateral recumbency under general anaesthesia using BD spinal needles into an EDTA tube and RNase-free Eppendorf tubes. For control dogs, blood samples were collected immediately prior to euthanasia, and CSF samples immediately after euthanasia.

***Profiled miRNAs***

The selected miRNAs for profiling were identified via a general literature search for eligible miRNAs [1–16]. For CSF samples let-7c, miR-16, miR-21, miR-24, mir-146a, miR-155, miR-181c and miR221-3p were profiled. For serum samples let-7a, let-7c, miR-15b, miR-16, miR-21, miR-23a, miR-24, miR-26a, miR-146a, miR-155, miR-181c and miR-221-3p were profiled.

***qPCR protocol***

One μL of each diluted cDNA sample was mixed with 5 μL 2X QuantiFast SYBR^®^Green PCR master mix (Qiagen), 2 μL RNase-free H2O and 1 μL of each primer (10 μM) in white 96-well PCR plates (ABgene^®^, Thermo Scientific, Denmark). Cycling conditions were set to 95 °C for 5 min, 40 cycles of 95 °C for 10 sec and 60 °C for 30 sec. A melting curve analysis was done at the end of the PCR to check for assay specificity.

***Manual curation of qPCR data***

The presence of contaminants in the cDNA synthesis was ruled out by visual detection of steady levels of *Caenorhabditis elegans* miR-39a spike across all samples. qPCR efficiencies were accepted between the range of 80-110%. Only samples with a Cq difference between replicates of ≤ 1.5 cycles (standard deviation (SD) on the Cq between replicates <1) were included for further analysis.

**References**

1. Gaitero L, Russell SJ, Monteith G, LaMarre J. Expression of microRNAs miR-21 and miR-181c in cerebrospinal fluid and serum in canine meningoencephalomyelitis of unknown origin. Vet J. 2016;216:122–4.

2. Sørensen SS, Nygaard A-B, Carlsen AL, Heegaard NH, Bak M, Christensen T. Elevation of brain-enriched miRNAs in cerebrospinal fluid of patients with acute ischemic stroke. Biomark Res. 2017;5:24.

3. Marioni-Henry K, Zaho D, Amengual-Batle P, Rzechorzek NM, Clinton M. Expression of microRNAs in cerebrospinal fluid of dogs with central nervous system disease. Acta Vet Scand. 2018;60:80.

4. Murugaiyan G, Garo LP, Weiner HL. MicroRNA-21, T helper lineage and autoimmunity. Oncotarget. 2015;6:9644.

5. Ma X, Zhou J, Zhong Y, Jiang L, Mu P, Li Y, et al. Expression, regulation and function of microRNAs in multiple sclerosis. Int J Med Sci. 2014;11:810.

6. Keller A, Leidinger P, Steinmeyer F, Stähler C, Franke A, Hemmrich-Stanisak G, et al. Comprehensive analysis of microRNA profiles in multiple sclerosis including next-generation sequencing. Mult Scler J. 2014;20:295–303.

7. Iborra M, Bernuzzi F, Invernizzi P, Danese S. MicroRNAs in autoimmunity and inflammatory bowel disease: crucial regulators in immune response. Autoimmun Rev. 2012;11:305–14.

8. Mycko MP, Cichalewska M, Machlanska A, Cwiklinska H, Mariasiewicz M, Selmaj KW. MicroRNA-301a regulation of a T-helper 17 immune response controls autoimmune demyelination. Proc Natl Acad Sci. 2012;109:E1248–57.

9. Garo LP, Murugaiyan G. Contribution of MicroRNAs to autoimmune diseases. Cell Mol Life Sci. 2016;73:2041–51.

10. Thamilarasan M, Koczan D, Hecker M, Paap B, Zettl UK. MicroRNAs in multiple sclerosis and experimental autoimmune encephalomyelitis. Autoimmun Rev. 2012;11:174–9.

11. Lescher J, Paap F, Schultz V, Redenbach L, Scheidt U, Rosewich H, et al. MicroRNA regulation in experimental autoimmune encephalomyelitis in mice and marmosets resembles regulation in human multiple sclerosis lesions. J Neuroimmunol. 2012;246:27–33.

12. Pauley KM, Cha S, Chan EK. MicroRNA in autoimmunity and autoimmune diseases. J Autoimmun. 2009;32:189–94.

13. Junker A, Krumbholz M, Eisele S, Mohan H, Augstein F, Bittner R, et al. MicroRNA profiling of multiple sclerosis lesions identifies modulators of the regulatory protein CD47. Brain. 2009;132:3342–52.

14. Sievers C, Meira M, Hoffmann F, Fontoura P, Kappos L, Lindberg RL. Altered microRNA expression in B lymphocytes in multiple sclerosis: towards a better understanding of treatment effects. Clin Immunol. 2012;144:70–9.

15. Sørensen SS, Nygaard A-B, Christensen T. miRNA expression profiles in cerebrospinal fluid and blood of patients with Alzheimer’s disease and other types of dementia–an exploratory study. Transl Neurodegener. 2016;5:6.

16. Zhu S, Pan W, Song X, Liu Y, Shao X, Tang Y, et al. The microRNA miR-23b suppresses IL-17-associated autoimmune inflammation by targeting TAB2, TAB3 and IKK-α. Nat Med. 2012;18:1077.
